# Supplementary material for: Linking neuron-axon-synapse architecture to white matter vasculature using high-resolution multimodal MRI in primate brain
Source: Imaging Neurosci (Camb). 2025 Jul 17;3:IMAG.a.77. doi: 10.1162/IMAG.a.77 (PMC12330855; doi:10.1162/IMAG.a.77)
Supplement: Supplementary Material [file IMAG.a.77_supp.pdf]

Supplementary Materials

Linking neuron-axon-synapse architecture to white matter vasculature using high-resolution multimodal MRI in primate brain

Authors

Ikko Kimura[1, 2], Takuya Hayashi[1, 3, *], Joonas A. Autio[1, 4, 5, *]

Affiliations

[1]Laboratory for Brain Connectomics Imaging, RIKEN Center for Biosystems Dynamics Research, Kobe, Japan

[2]Neurophysics Group, Danish Research Centre for Magnetic Resonance, Copenhagen University Hospital - Hvidovre and Amager, Copenhagen, Denmark

[3]Department of Brain Connectomics, Kyoto University Graduate School of Medicine, Kyoto, Japan

[4]Department of Neuroscience, Washington University in St. Louis, St. Louis, MO, USA

[5]Department of Radiology, Washington University in St. Louis, St. Louis, MO, USA

***Corresponding authors**

Joonas A. Autio, Ph.D.

Department of Neuroscience

Washington University in St. Louis

4370 Duncan Ave, St. Louis, MO 63110, USA

E-mail: autio@wustl.edu

Takuya Hayashi, M.D., Ph.D.

Laboratory for Brain Connectomics Imaging

RIKEN Center for Biosystems Dynamics Research

6-7-3 MI R&D Center 3F, Minatojima-minamimachi

Chuo-ku, Kobe 650-0047, Japan

E-mail: takuya.hayashi@riken.jp

Contains Supplementary Figures 1−5 and Table 1

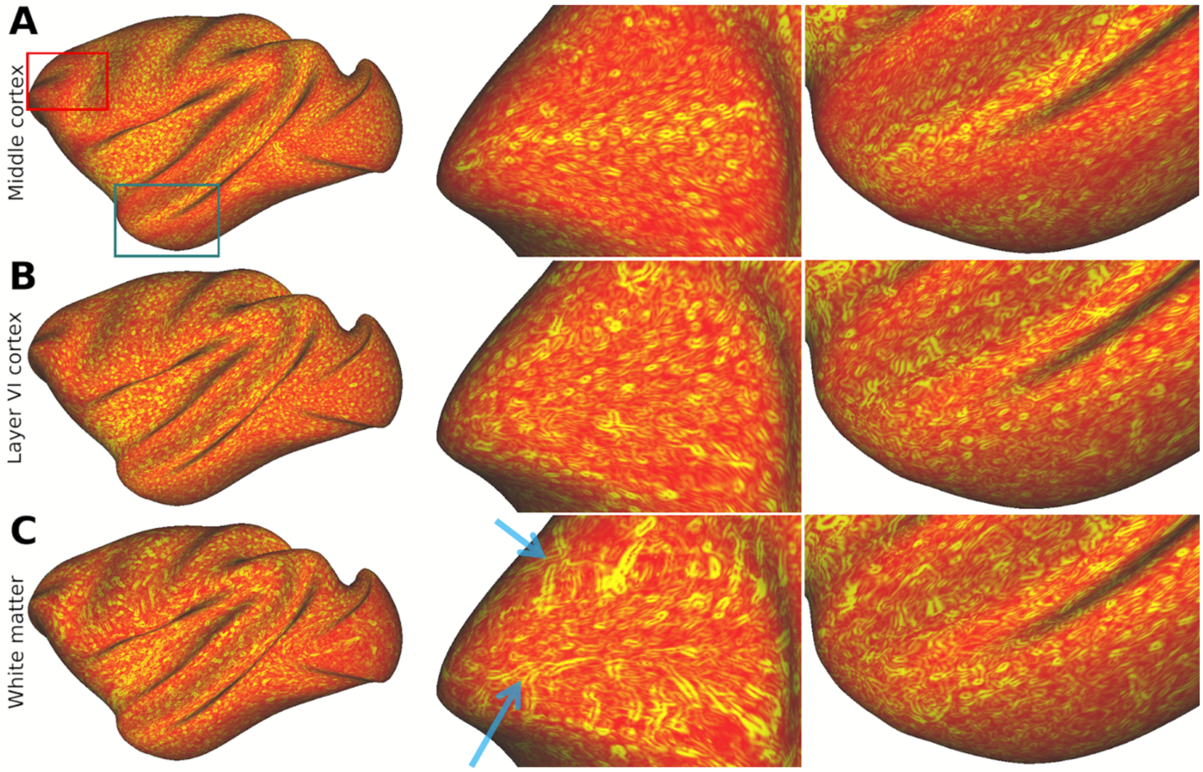

Supplementary Figure 1. Regional variations in superficial white matter macrovasculature patterns across the brain. Spatial gradients of the ferumoxytol-weighted image are shown for the equivolumetric layers (EL): (A) EL4a, located near the cortical midthickness, and (B) EL6b, situated adjacent to the white matter surface. (C) An equidistant superficial white matter surface located beneath the cortical gray matter. Distinct and variable vessel orientations were observed in the prefrontal cortex (red box; middle panels), whereas subtler variations in vessel orientation were evident near the temporal pole (blue box; right panels). Blue arrows indicate exemplar vessels oriented orthogonal to each other.

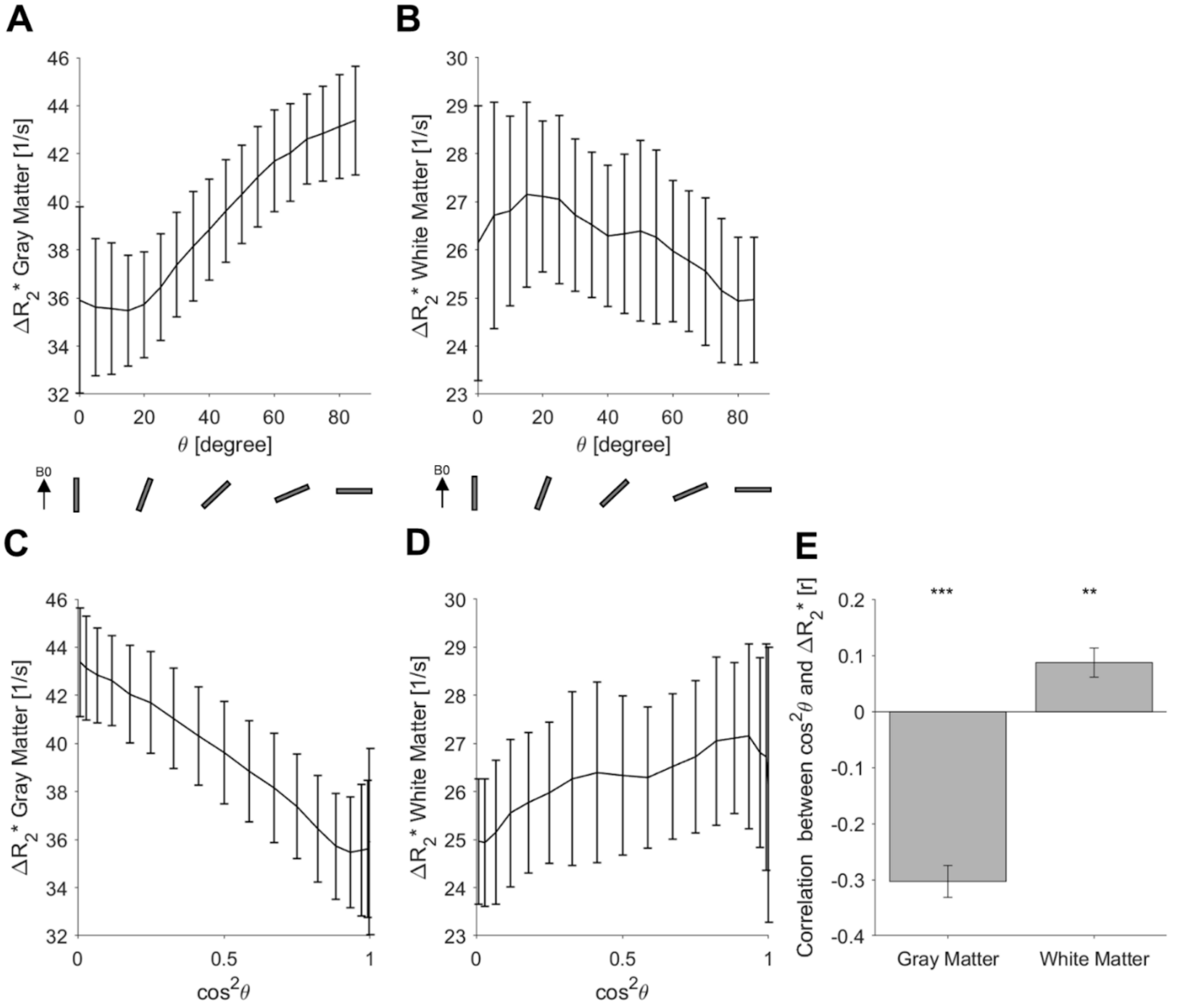

Supplementary Figure 2. Relationship between ferumoxytol-induced change in transverse relaxation rate (ΔR_2*) and the angle (θ) between the normal of cortex and B_0. **(A)** Relationship between ΔR_2* and θ in the cortical gray matter. **(B)** Relationship between ΔR_2* and θ in the superficial white matter. **(C)** Relationship between ΔR_2* and $cos^2\theta$ in the cortical gray matter. **(D)** Relationship between ΔR_2* and $cos^2\theta$ in the superficial white matter. **(E)** Pearson's correlation coefficients between ΔR_2* and the $cos^2\theta$ within the cortical gray matter (left) and superficial white matter (right). Linear relationship is more prominent in the cortical gray matter probably due to more systematic orientation of large vessels. ** $P < 0.005$ and ** $P < 0.001$ (Bonferroni-corrected).

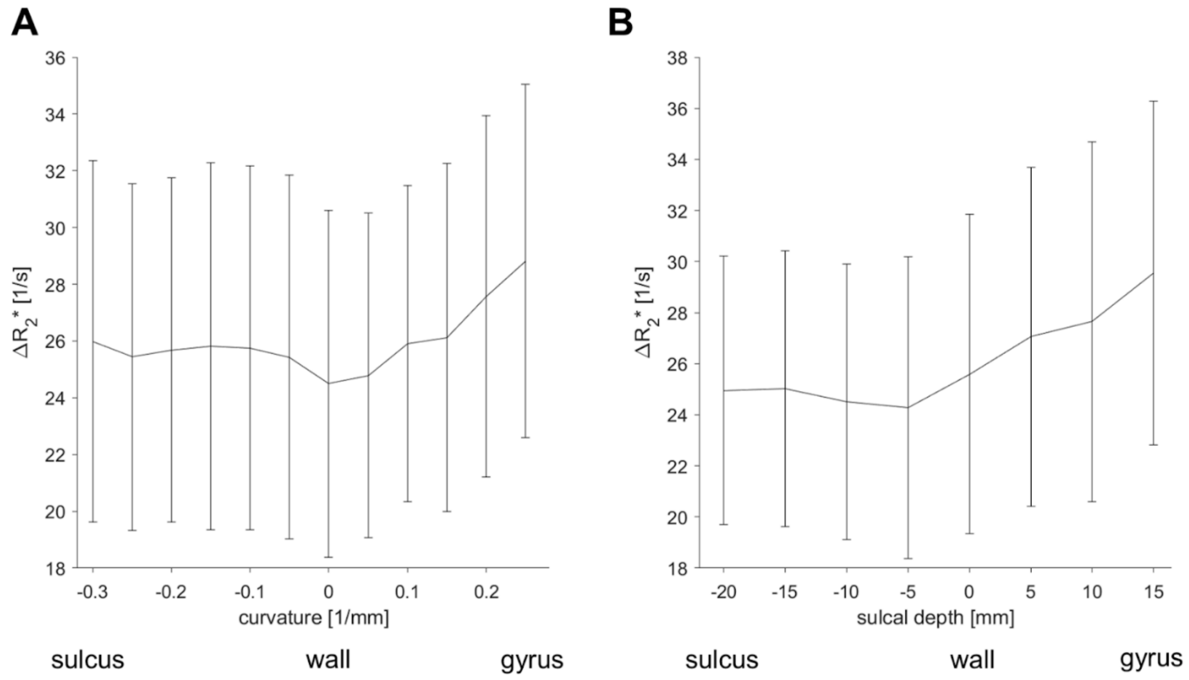

Supplementary Figure 3. Relationship between superficial white matter vasculature and cortical geometry. Ferumoxytol-induced change in transverse relaxation rate **(**ΔR_2*) plotted as a function of **(A)** curvature and **(B)** sulcal depth. In both plots, negative values correspond to sulcus and positive values indicate gyrus.

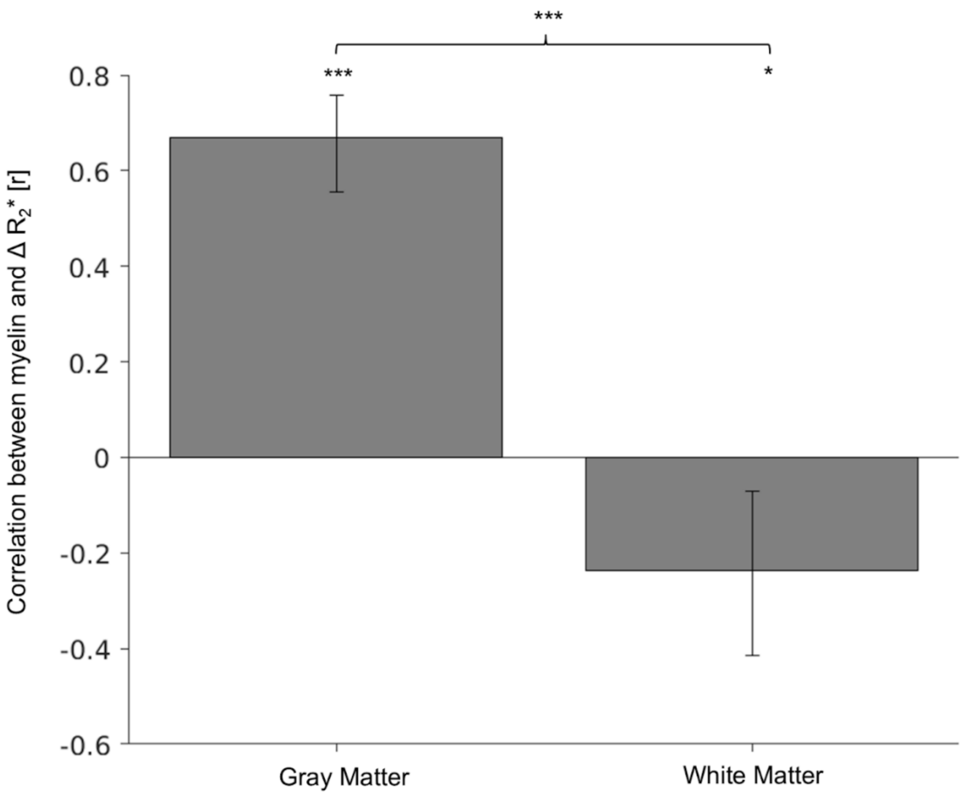

Supplementary Figure 4. Contrasting relationship between blood volume and myelin across cortical gray matter and superficial white matter. Correlation between ΔR_2*, an indirect proxy measure of vascular volume, and T1w/T2w-FLAIR, an indirect proxy measure of myelin density. Data were parcellated using the M132 macaque atlas (See Methods). ** *P* < 0.005 and ** *P* < 0.001 (Bonferroni-corrected).

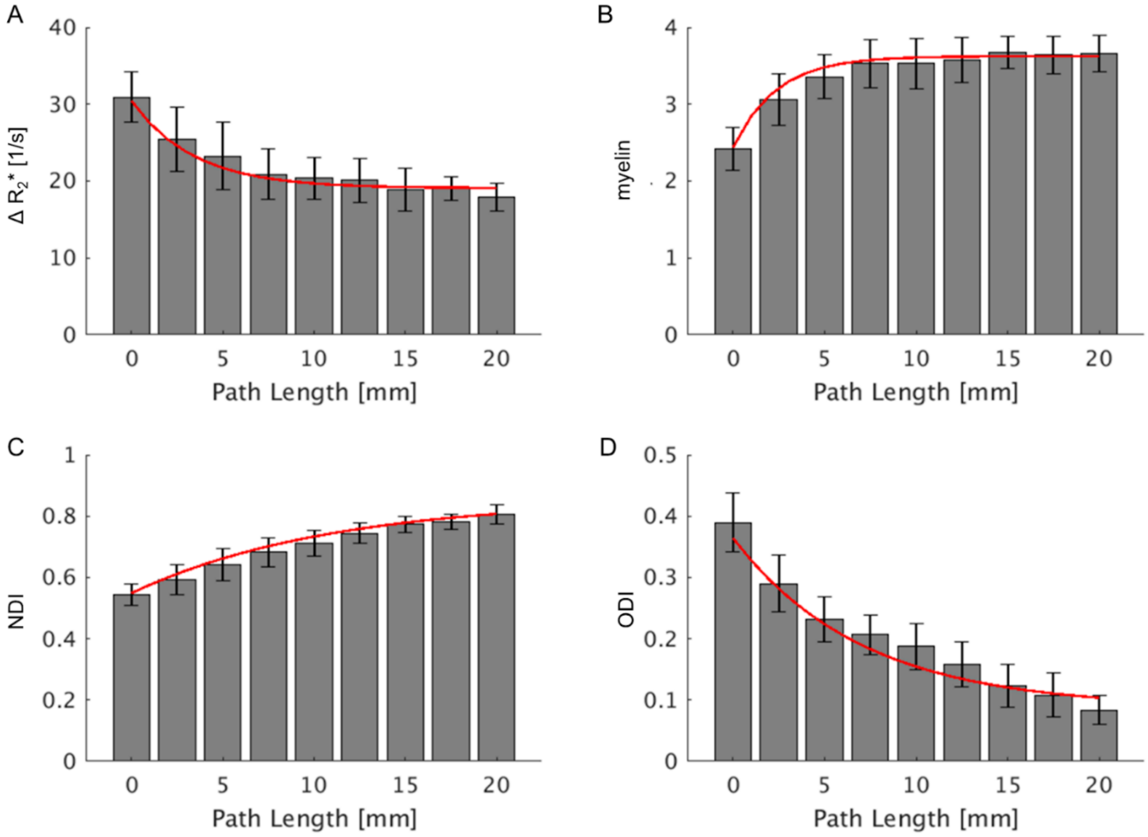

Supplementary Figure 5. Relationships between white matter vascularity, microstructure, and brain geometry. The relationships between path length from superficial white matter and **(A)** ferumoxytol-induced change in transverse relaxation rate (ΔR_2*), **(B)** T1w/T2w-FLAIR, an indirect proxy measure of myelin density, **(C)** neurite density index (NDI), and **(D)** orientation dispersion index (ODI).

	Exponential	Linear	Quadratic	Logistic
ΔR_2*				
AICc	**1717**	1804	1725	1718
BIC	**1728**	1811	1736	1733
NDI				
AICc	**-1086**	-1061	-1085	-1085
BIC	**-1075**	-1053	-1073	-1070
ODI				
AICc	**-1172**	-1112	-1156	-1171
BIC	**-1161**	-1104	-1145	-1156

Table S1. Model comparison results for fitting each metric as a function of relative distance. Values in bold indicate the lowest AICc and BIC scores across models, representing the best-fitting model for each metric. Abbreviations: ΔR_2*: transverse relaxation rate; NDI: neurite density index; ODI: orientation dispersion index; AICc: corrected Akaike Information Criterion; BIC: Bayesian Information Criterion.
